# No evidence of abnormal metabolic or inflammatory activity in the brains of patients with rheumatoid arthritis: results from a preliminary study using whole-brain magnetic resonance spectroscopic imaging (MRSI)

**DOI:** 10.1007/s10067-019-04923-5

**Published:** 2020-01-30

**Authors:** Christina Mueller, Joanne C. Lin, Halle H. Thannickal, Altamish Daredia, Thomas S. Denney, Ronald Beyers, Jarred W. Younger

**Affiliations:** 1 Department of Psychology, University of Alabama at Birmingham, Birmingham, AL USA; 2 School of Pharmacy, Faculty of Medical and Health Sciences, University of Auckland, Auckland, New Zealand; 3 Department of Biology, University of Michigan, Ann Arbor, MI USA; 4 Department of Electrical and Computer Engineering, Auburn University, Auburn, AL USA

**Keywords:** Brain metabolites, Brain temperature, Magnetic resonance spectroscopy, Rheumatoid arthritis

## Abstract

**Introduction/objectives:**

Many individuals with rheumatoid arthritis (RA) report persistent fatigue even after management of peripheral disease activity. This study used whole-brain magnetic resonance spectroscopic imaging (MRSI) to investigate whether abnormal inflammatory activity in the central nervous system may be associated with such symptoms. We hypothesized that RA patients would show higher brain choline (CHO), myo-inositol (MI), and lactate (LAC), and higher brain temperature than healthy controls. We further hypothesized that the metabolite levels would be positively correlated with self-reported fatigue.

**Method:**

Thirteen women with RA provided fatigue severity ratings and underwent whole-brain MRSI and a joint examination. Thirteen healthy controls (HC) provided comparison imaging and fatigue data. CHO, MI, LAC, and brain temperature in 47 brain regions were contrasted between groups using independent-samples *t* tests. Significant differences were determined using a false discovery rate (FDR)-adjusted *p* value threshold of ≤ 0.0023. Secondary analyses obtained correlations between imaging and clinical outcomes in the RA group.

**Results:**

No brain metabolic differences were identified between the groups. In the RA group, fatigue severity was positively correlated with CHO in several brain regions—most strongly the right frontal lobe (r_s_ = 0.823, *p* < 0.001). MI was similarly correlated with fatigue, particularly in the right calcarine fissure (r_*s*_ = 0.829, *p* < 0.001). CHO in several regions was positively correlated with joint swelling and tenderness.

**Conclusions:**

We conclude that abnormal brain metabolites are not a common feature of RA, but may been seen in patients with persistent fatigue or disease activity after conventional treatment.

**Table:** 

**Key Points** *• Whole-brain magnetic resonance spectroscopy revealed no metabolic abnormalities in the brain in patients with rheumatoid arthritis.* *• Brain choline levels were correlated with fatigue severity reported by RA patients and with peripheral joint swelling and tenderness.* *• Brain myo-inositol levels were similarly correlated with fatigue severity in RA patients.*

**Electronic supplementary material:**

The online version of this article (10.1007/s10067-019-04923-5) contains supplementary material, which is available to authorized users.

## Introduction

Rheumatoid arthritis (RA) is a peripheral autoimmune inflammatory disorder affecting the joints. It is characterized by synovial inflammation leading to joint damage, production of peripheral inflammatory cytokines such as tumor necrosis factor α (TNF-α), and chronic immune system activation [1, 2]. Although neurological exams are typically normal, RA patients often complain of symptoms such as fatigue that suggest involvement of the central nervous system (CNS). Half of patients with RA experience significant fatigue even while their joint disease activity is adequately controlled with medications [3, 4]. It is possible that the ongoing fatigue in RA is due to inflammatory activity in the brain that cannot be addressed by conventional RA medications not designed to cross the blood-brain barrier (BBB).

High levels of several peripheral pro-inflammatory cytokines, including interleukin (IL)-1, IL-6, and TNF-α, are present in the blood of RA patients [5–7]. These cytokines may enter the brain via a compromised BBB and activate microglia [8–10]. When activated, microglia increase the production of the same pro-inflammatory factors (e.g., IL-1, IL-6, and TNF-α) as those seen in systemic circulation [11]. When active chronically, this pro-inflammatory activity in the brain can manifest behaviorally as fatigue, cognitive problems, and mood abnormalities [12]. Supporting this idea, Bodnar and colleagues demonstrated that inducing synovial inflammation in an animal model of RA is followed by increases in pro-inflammatory cytokine levels in the brain [13].

There is currently no available technique to directly measure levels of inflammatory cytokines in the brains of living humans. Non-invasive neuroimaging tools such as MRSI and positron emission tomography (PET) have been utilized to demonstrate elevated neuroinflammatory markers in other diseases involving significant fatigue of a suspected central origin, such as chronic fatigue syndrome and fibromyalgia [14–16]. While MRSI is limited to only a few measurable metabolites, several of them have been linked to inflammatory activity in the brain. Those metabolites include choline (CHO), a marker of cell turnover, myo-inositol (MI), a marker of glial proliferation, and lactate (LAC), a marker of anaerobic cell metabolism. Additionally, MRSI can yield absolute brain temperature, which may be elevated in cases of excess metabolic demand due to neuroinflammation.

In the current study, we examined whole-brain metabolite and temperature abnormalities in RA. RA participants and matched healthy controls (HC) underwent an MRSI session that allows metabolites to be measured in thousands of voxels across the brain. Those levels were contrasted between RA and HC groups. Our primary hypothesis was that the RA group would show elevated brain CHO, MI, LAC, and temperature. Our secondary hypothesis was that fatigue severity in the RA group would be positively correlated with CHO, MI, LAC, and temperature. Confirmation of neurochemical abnormality in the brain in RA could indicate neuroinflammation and help to explain why these individuals have significant fatigue that is not alleviated by conventional rheumatologic treatments.

## Materials and methods

### Participants

All study procedures were approved by the UAB Institutional Review Board and have been performed in accordance with the ethical standards laid down in the 1964 Declaration of Helsinki and its later amendments. Study advertisements were distributed at the University of Alabama at Birmingham (UAB) campus, waiting areas of local physician offices, and the laboratory’s e-mail newsletter. Interested individuals contacted the laboratory via phone or e-mail and were preliminarily screened for inclusion criteria by the research team.

Participants with RA met the following inclusion criteria: (i) aged 18–65 years; (ii) physician diagnosis of adult-onset RA; (iii) currently receiving pharmacological treatment for RA with disease-modifying anti-rheumatic drugs and/or biologic agents; (iv) average self-reported daily pain or fatigue of ≥ 6 on an 11-point scale.

Control participants were also aged between 18 and 65, were matched within 2 years of participants with RA, did not have a history of rheumatoid arthritis or other autoimmune disorders, and reported average daily pain and fatigue of ≤ 2 on an 11-point scale.

All potential participants in the RA or HC groups were excluded for (i) MRI safety contraindications; (ii) diagnosed neurological, major psychiatric, or inflammatory disorders; (iii) psychostimulant use; (iv) smoking; or (v) regular use of non-steroidal anti-inflammatory drugs (NSAIDs; e.g., ibuprofen, naproxen, aspirin). Occasional use of NSAIDs was permitted, but participants were asked not to use them within 24 h of study participation. Medical history and medication use were self-reported; medical records were not reviewed for this study. Information on marital status and education was not collected. Participants gave written informed consent prior to inclusion in the study.

### Study protocol

Study sessions lasted approximately 2 h. Participants completed demographic and symptom questionnaires before neuroimaging. Body temperature was measured binaurally with a Braun Pro 4000 ThermoScan thermometer before and after MRI scans. Additionally for the RA group, a physical joint examination was performed according to the Modified Disease Activity Scores (DAS-28) [17] protocol to provide a measure of peripheral pain sensitivity. Conventional DAS-28 scores incorporating a measure of peripheral inflammatory activity (e.g., erythrocyte sedimentation rate, c-reactive protein) were not available as no blood tests were performed, so a validated marker of total disease activity was not collected.

Participants also self-reported current disease activity on a visual analogue scale in response to the prompt, “How active was your arthritis during the past week?”, with minimum and maximum anchor points reading “not active at all” and “extremely active”, respectively. Participants were compensated $150 via check following study completion.

### Symptom questionnaires

The Fatigue Severity Scale (FSS) [18] and Hospital Anxiety and Depression Scales (HADS) [19] were administered in order to quantify the severity of RA-associated symptoms. The nine-item FSS assesses fatigue intensity and functional disability on a seven-point Likert scale ranging from 1 (“strongly disagrees”) to 7 (“strongly agrees”). The HADS contains two subscales of seven items each on a four-point (0–3) scale, measuring anxiety (HADS-A) and depressed mood (HADS-D) over the past week.

### Neuroimaging acquisition

MRSI data were acquired on a 3.0T Siemens Magnetom Prisma (Siemens Medical Solutions, Erlangen, Germany) with 20-channel head coil. Whole-brain MRSI data were acquired with a 3D echo-planar spectroscopic imaging (EPSI) sequence [20] with the following parameters: TR, 1550 ms; TE, 17.6 ms; lipid inversion nulling with inversion time (TI), 198 ms; spin-echo excitation of a 135-mm axial slab covering the cerebrum; flip angle, 71°; FOV, 280 × 280 × 180 mm; matrix, 50 × 50 × 18; GRAPPA factor, 1.3; voxel resolution = 5.6 × 5.6 × 10 mm.

For anatomical reference, a T1-weighted image covering the cranium was acquired using a magnetization prepared rapid gradient echo (MPRAGE) sequence with the following parameters: repetition time (TR), 2000 ms; echo time (TE), 2.51 ms; flip angle, 8°; 208 slices; slice thickness, 0.9 mm; field of view (FOV) = 230 × 230 mm; matrix = 256 × 256; voxel resolution, 0.9 × 0.9 × 0.9 mm.

To detect cases of abnormal cerebral blood flow, a 2D arterial spin labeling (ASL) image was acquired with PICORE labeling and the following parameters: TR, 2500 ms; TE, 16.18 ms; TI = 1800 ms; 12 slices; FOV = 256 × 256 mm; matrix = 64 × 64 mm; voxel resolution = 4 × 4 × 8 mm. Sixty pairs of label/control images were collected in the axial plane.

### MRSI processing

MRSI data were processed using the Metabolite Imaging Data Analysis System (MIDAS) software [21]. The following were applied during preprocessing: spatial reconstruction, B0 shift correction, interpolation to 64 × 64 × 32 points, and smoothing with Gaussian kernel (5 mm in plane, 7 mm through plane). The FITT2 module was used to carry out spectral fitting with Gaussian line shapes. Metabolite maps were normalized to institutional units using tissue water from an interleaved non-water-suppressed MRSI acquisition. Images were registered to each subject’s high-resolution T1-weighted image and the tissue distribution for spectroscopic images was determined following segmentation of the T1-weighted images with FSL/FAST [22]. A non-linear spatial transform was applied to the metabolite maps and tissue distribution images for registration to 2 mm Montreal Neurological Institute (MNI) space. A modified version of the Automated Anatomical Labeling (AAL) atlas [23] was used to delineate 47 regions of interest (ROIs) for statistical comparisons between groups.

### Data analysis

**Fig. 1:**
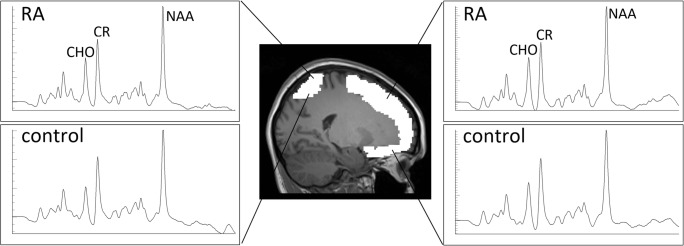
Representative integrated spectra in the right frontal and parietal lobes from a participant in the RA group (top panels) and control group (bottom panels). X axis = parts per million. Y axis = institutional units.

MRS data were analyzed using the Project Review and Analysis (PRANA) and Map Integrated Spectrum (MINT) modules within MIDAS. Voxels were excluded based on the following criteria: (i) fitted metabolite linewidth above 13 Hz; (ii) outlying values 2.5 standard deviations above or below the mean of all valid values within the image; (iii) a Cramér-Rao Lower Bound (CRLB) for creatine (CR) fitting above 40%; and (iv) greater than 30% contribution from cerebrospinal fluid to the voxel volume. Atlas-defined ROIs were inversely transformed into subject space, and spectral averaging was performed over the ROIs to obtain CHO, MI, NAA, LAC, and CR values (area under curve, AUC) in each region. Representative integrated spectra in the right frontal and parietal cortices for one participant from the RA and control group are shown in Fig. 1. Metabolites were expressed as ratios relative to CR to allow for comparisons with other studies. Brain temperature was calculated based on the metabolite maps using the following formula: T = − 102.61 × Δ(ƒ_H2O_-ƒ_CR_) + 206.1 °C where ƒ_H2O_ and ƒ_CR_ represent the resonant frequencies of the water and CR peaks, respectively [24].

ASL data were processed with ASLtbx [25] for SPM12 (Wellcome Trust Centre for Neuroimaging, London, UK) within MATLAB (MathWorks, Natick, MA, USA). Images were realigned to the mean of all control images and smoothed with a 3D 6-mm Gaussian kernel. Control images were subtracted from each corresponding labeled image for cerebral blood flow (CBF) quantification. Difference images were averaged and the mean CBF across all voxels over the brain was quantified for each participant.

### Statistics

Main analyses used univariate independent-samples *t* tests to compare metabolite ratios and brain temperature in each region of interest between RA patients and healthy controls. The following were the dependent variables: (i) CHO/CR; (ii) MI/CR; (iii) LAC/CR; (iv) NAA/CR; and (v) brain temperature. Tests were considered significant at a false discovery rate (FDR)-adjusted *p* value of ≤ 0.0023.

As a secondary analysis, the relationship between MRSI outcomes and clinical outcomes was assessed. Spearman’s rho (r_s_) correlation coefficients were calculated between each MRSI outcome and self-reported fatigue severity. In addition, to determine if peripheral disease activity was associated with MRSI findings, the outcomes were correlated with joint swelling and tenderness. The correlations were tested (two-tailed) using the FDR-corrected (*p* ≤ 0.0023) threshold.

To test for any differences in cerebral perfusion, independent-samples *t* tests were used, contrasting whole-brain average cerebral blood flow between groups.

## Results

### Participants

Eighteen women with RA were enrolled in the study. Sixteen healthy women were recruited and age-matched to participants in the RA group who had provided usable data.

Data from three RA participants were excluded due to visible movement during acquisition, and data from two RA participants were excluded due to failure to meet initial quality criteria (less than 40% accepted voxels). The final dataset contained data from 13 women with RA. Data from HC participants were lost due to failure to meet initial quality criteria (*n* = 1), lack of age match (*n* = 1), and failure to complete the protocol (*n* = 1), leaving data from 13 healthy age-matched controls. One participant from each group was left-handed, and the remaining participants were right-handed. Three women from each group were post-menopausal, while the remaining ten had not entered menopause.

Group means and results from independent *t* tests comparing participant characteristics are shown in Table 1. There was no difference between RA patients’ and controls’ mean age, body temperature (average tympanic temperature measured before and after imaging), and global cerebral blood flow. As expected, the RA group had higher average fatigue severity, anxiety, and depressed mood, than the HC group. Depressive symptoms in the RA group were below clinically significant levels (mean = 5.62), with 11 or higher indicating moderate depression severity [26].

**Table 1:** Group means and their standard deviations (SD) for clinical and questionnaire data. *p* values refer to independent-samples *t* tests comparing the group means. Median values are reported alongside the group means

	RA group (*n* = 13)		Control group (*n* = 13)		
	Mean (SD)	Median	Mean (SD)	Median	*p*
Age (years)	40.92 (10.79)	38.00	40.85 (11.84)	38.00	0.986
Fatigue severity (9–63)	6.92 (1.85)	7.00	0.69 (0.85)	0.00	< 0.001
Anxiety (0–21)	8.31 (3.45)	8.00	3.08 (2.84)	4.00	< 0.001
Depression (0–21)	5.62 (3.57)	5.00	0.85 (1.52)	0.00	< 0.001
DAS-28 swollen joint count (0–28)	4.69 (5.85)	2.00			
DAS-28 tender joint count (0–28)	5.31 (6.49)	2.00			
Average body temperature (°C)	97.94 (0.59)	97.80	98.33 (0.54)	98.33	0.084
Global CBF (ml/100 g/min)	59.95 (10.59)	60.86	60.83 (13.76)	56.92	0.857

Three RA participants (23%) were receiving treatment with biologic DMARDs only, two (15%) were being treated with synthetic DMARDs (methotrexate, hydroxychloroquine) only, and seven (54%) were receiving both. Three participants were additionally being actively treated with corticosteroids. Six were also taking opioid pain medication. A complete list of medications can be found in Online Resource 1.

## Main results

**Fig. 2:**
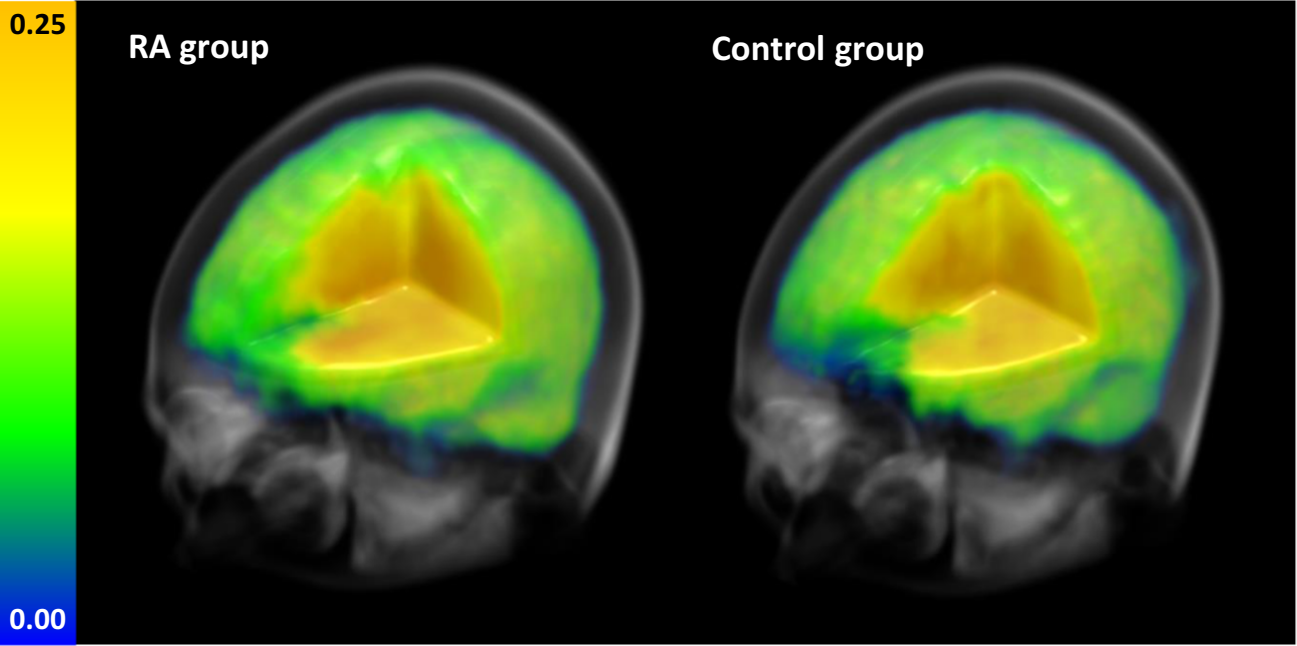
Mean CHO/CR ratios across the brain in the RA and control group. There were no significant differences in CHO/CR in any of the assessed regions of interest

There were no significant MRSI outcome differences between groups for any brain region at the *p* < 0.0023 threshold. Maps of the average CHO/CR ratios in the RA and control group are visualized in Fig. 2. Results for all group contrasts can be found in Online Resource 2.

### Secondary analyses (RA group only)

**Fig. 3:**
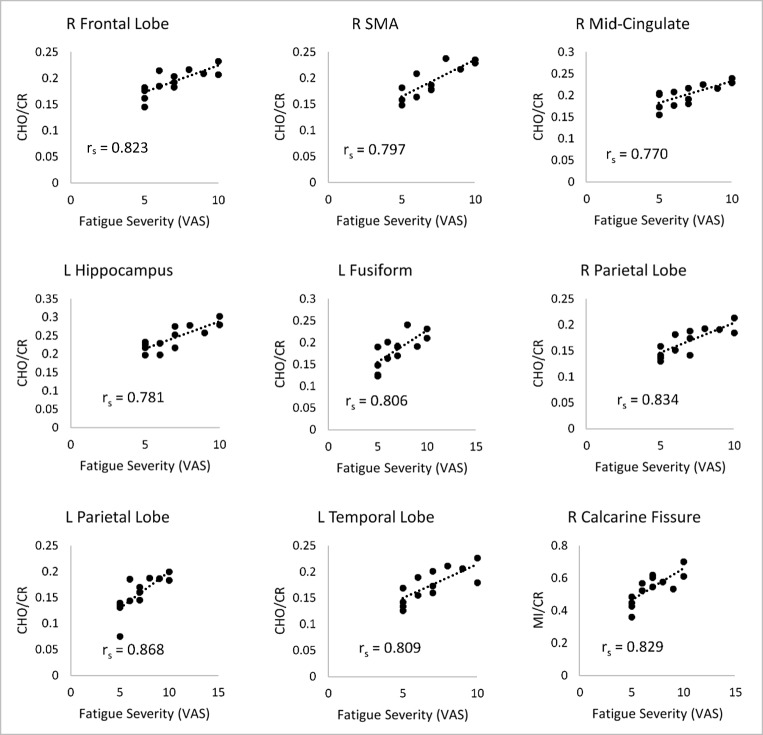
Significant Spearman correlations between metabolite ratios and fatigue severity on the visual analog scale. The correlations are significant at a false discovery rate-corrected threshold of *p* < 0.0023. CHO, choline; CR, creatine; L, left; R, right; SMA, supplementary motor area; VAS, visual analog scale; MI, myo-inositol

Spearman correlation coefficients between fatigue and CHO/CR and MI/CR are displayed in Table 2 and Fig. 3. Self-reported fatigue was positively correlated with CHO/CR and MI/CR in several regions at the FDR-corrected threshold of *p* < 0.0023. Fatigue was significantly correlated with CHO/CR in the: right frontal lobe (r_s_ = 0.823), right supplemental motor area (r_s_ = 0.797), right mid-cingulate (r_s_ = 0.770), left hippocampus (r_s_ = 0.781), left fusiform gyrus (r_s_ = 0.806), right parietal lobe (r_s_ = 0.834), left parietal lobe (r_s_ = 0.868), and left temporal lobe (r_s_ = 0.809). Fatigue was significantly correlated with MI/CR in the right calcarine fissure (r_s_ = 0.829).

**Table 2:** Spearman correlations between metabolite ratios and temperature in regions of interest and clinical outcome data in RA patients. Results surviving false discovery rate corrections (equivalent to uncorrected *p* < 0.0023) are shown in italic font

	CHO/CR			MI/CR		
ROI	FSS VAS	DAS-28 swollen	DAS-28 tender	FSS VAS	DAS28 swollen	DAS28 tender
Precentral R	0.716**	0.782**	0.678*	0.705**	0.048	0.126
Precentral L	0.742**	0.660*	0.764**	0.452	0.564*	0.299
Frontal lobe R	0.823***	0.629*	0.683*	0.413	− 0.111	− 0.081
Frontal lobe L	0.674*	0.672*	0.759**	0.615*	0.425	0.156
Rolandic Oper R	0.708**	0.799**	0.653*	0.427	− 0.034	− 0.033
Rolandic Oper L	0.714**	0.700**	0.709**	0.309	0.071	0.321
Supp motor area R†	0.797**	0.643*	0.624*	0.157	0.320	− 0.190
Supp motor area L	0.686**	0.598*	0.672*	0.627*	0.235	0.131
Insula R	0.590*	0.666*	0.773**	0.211	− 0.111	− 0.360
Insula L	0.424	0.760**	0.658*	0.177	− 0.190	− 0.354
Anterior cingulum R†	0.121	0.440	0.480	− 0.420	− 0.403	− 0.275
Anterior cingulum L	0.562*	0.385	0.619*	0.051	− 0.184	− 0.268
Mid cingulum R	0.770**	0.485	0.717**	− 0.093	− 0.193	− 0.379
Mid cingulum L	0.635*	0.581*	0.778**	− 0.084	− 0.340	− 0.379
Posterior cingulum R†	0.395	0.547	0.603*	0.553	0.393	0.296
Posterior cingulum L	0.166	0.539	0.544	0.466	0.601*	0.488
Hippocampus R	0.590*	0.473	0.709**	0.509	0.357	0.198
Hippocampus L	0.781**	0.360	0.460	0.323	0.346	0.011
Calcarine R	0.753**	0.779**	0.661*	0.829***	0.419	0.307
Calcarine L	0.548	0.734**	0.781**	0.731**	0.269	0.257
Cuneus R	0.705**	0.709**	0.488	0.613*	0.400	0.179
Cuneus L	0.596*	0.689**	0.516	0.596*	0.337	0.008
Lingual gyrus R	0.753**	0.768**	0.756**	0.320	0.476	0.265
Lingual gyrus L	0.618*	0.743**	0.664*	0.492	0.561*	0.360
Occipital lobe R	0.705**	0.726**	0.633*	0.652*	0.439	0.293
Occipital lobe L	0.764**	0.677*	0.650*	0.756**	0.309	0.112
Fusiform gyrus R	0.761**	0.745**	0.709**	0.545	0.473	0.165
Fusiform gyrus L	0.806***	0.615*	0.695**	0.427	0.346	0.153
Postcentral gyrus R	0.672*	0.791**	0.736**	0.705**	0.541	0.527
Postcentral gyrus L	0.694**	0.714**	0.820***	0.582*	0.660*	0.455
Parietal lobe R	0.834***	0.706**	0.683*	0.683*	0.476	0.268
Parietal lobe L	0.868***	0.675*	0.678*	0.672*	0.281	0.095
Precuneus R	0.663*	0.700**	0.692**	0.627*	0.504	0.240
Precuneus L	0.551	0.706**	0.770**	0.320	0.332	− 0.067
Paracentral lobule R	0.705*^a^	0.519^a^	0.628* ^a^	0.342 ^a^	0.312 ^a^	− 0.018 ^a^
Paracentral lobule L	0.312	0.703**	0.695**	0.247	0.453	0.011
Caudate R	0.278	0.283	0.354	0.205	− 0.230	− 0.293
Caudate L	0.407	0.417	0.455	0.253	− 0.023	− 0.036
Putamen R	0.014	0.221	0.449	0.424	0.077	0.089
Putamen L	0.601*	0.567*	0.611*	0.444	0.587*	0.237
Pallidum R	0.292	0.196	0.533	0.441	0.332	0.050
Pallidum L	0.545	0.402	0.477	0.674*	0.632*	0.290
Thalamus R	0.556*	0.734**	0.658*	− 0.037	0.009	− 0.156
Thalamus L	0.573*	0.451	0.304	0.388	0.315	0.139
Temporal lobe R	0.745**	0.856***	0.695**	0.480	0.453	0.232
Temporal lobe L	0.809***	0.731**	0.678*	0.399	0.128	− 0.006
Cerebellum	0.745**	0.686**	0.725**	0.565*	0.057	− 0.011

*VAS* visual analog scale

*p < 0.05; **p < 0.01; ***p < 0.001

*n* = 13 (unless an exception is noted)

^a^*n* = 12

CHO/CR in several brain regions was positively correlated with DAS-28 joint swelling or tenderness (see Table 2 for details), including the right precentral gyrus, right rolandic operculum, right insula, left mid-cingulate, right calcarine fissure, left calcarine fissure, right lingual gyrus, right postcentral gyrus, left postcentral gyrus, left precuneus, and right temporal lobe. MI/CR was not associated with joint swelling or tenderness.

There were no significant associations between clinical outcomes and LAC/CR, NAA/CR, or brain temperature. A table displaying these associations is included in Online Resource 3.

## Discussion

In this study, we used MRSI to examine markers of brain inflammation in physician-managed RA patients with ongoing fatigue. Thirteen RA patients were contrasted with thirteen age- and gender-matched healthy controls. There was no significant evidence of elevated inflammatory markers in any brain regions of the RA group.

The lack of evidence for brain inflammation in RA is consistent with the small number of similar studies conducted previously. To our knowledge, only one previous study has examined brain levels of CHO, LAC, or NAA in RA versus healthy controls. Emmer and colleagues [27] reported no significant differences between RA patients and controls in CHO, LAC, or NAA when performing single-voxel spectroscopy in the centrum semiovale. We are also aware of only one PET study using a translocator protein (TSPO) radioligand to examine possible brain inflammation in RA versus controls. Forsberg and colleagues [44] used the TSPO radioligand 11C-PBR28 in 15 RA patients and 15 healthy controls. They found no differences in TSPO uptake between the groups. To date, therefore, there is no direct evidence that brain inflammation is a typical aspect of RA.

Despite seeing no group differences in brain metabolite levels, we observed several significant correlations between brain metabolite levels and self-reported fatigue. The fatigue and CHO correlation was greater than 0.7 in 21 out of the 47 regions tested. CHO levels were similarly positively correlated with both the DAS-28 swollen joint and tender joint counts. These results are similar to the earlier spectroscopic study that found brain CHO to be positively correlated with erythrocyte sedimentation rate in RA [27]. These results suggest that higher CHO levels throughout the brain may be meaningfully associated with RA disease severity, and may contribute to fatigue severity. However, it is difficult to determine the importance of brain CHO in RA given the lack of abnormal levels in the group overall.

We previously reported elevated CHO, LAC, MI, and temperature throughout the brain in myalgic encephalomyelitis/chronic fatigue syndrome (ME/CFS) [14]. Despite the fact that both the ME/CFS group in the previous study and RA group in the current study had significant self-reported fatigue (≥ 6 out of 10), only the ME/CFS group evidenced signs of brain inflammation. The difference in results could indicate ME/CFS is a CNS disease, while RA is a disease primarily of the peripheral joints. The MRSI scan may, therefore, be useful in identifying individuals with central inflammatory origins of fatigue.

There are a few limitations to this study. First, while the FDR-corrected *p* value helps to control for false positives, it also increases the chances of false negatives, especially when used with a small sample size. There were several tests that were significant at a *p* < 0.05 level that were not discussed, but may represent true group differences when studied with a greater sample size. This problem can be addressed only with repeating the study in a larger sample. However, we note that we were able to identify several group differences using a similar sample size, and the same scanning parameters and analysis techniques, in a previous study of ME/CFS and healthy controls [14]. The small sample size also limits generalizability, and leaves out important RA subgroups. Larger sample sizes would allow analyses to be conducted on peripheral disease severity, inflammatory markers, and medication use. It is possible that individuals not receiving medication or other adequate medical care would show more severe brain alterations. It should also be noted that psychotropic medications in the RA group could have affected brain metabolite levels and temperature. Patients were allowed to continue taking their regularly prescribed medications to minimize the effects of acute withdrawal on brain metabolites or temperature [28]. Duloxetine, for example, has been shown to affect hypothalamic thermoregulation [29], and hippocampal NAA [30]. Benzodiazepines and non-benzodiazepine sedative-hypnotics can change body temperature in animal models [31, 32] and brain glutamine synthesis [33], but effects on brain temperature, NAA, CHO, or MI have not been reported [34]. Opioids similarly have been shown to affect brain glutamate in reward system areas [35–38], but not NAA, CHO, or MI. Two human studies have found reduced NAA in frontal gray matter in opioid addicted individuals [39, 40]. Several animal studies have reported temperature changes in the nucleus accumbens following intravenous opioid administration [41–43]. Therefore, there is the potential for a medication confound in the present study. However, the potential confound is minimized by the low number of participants taking each medication class (six taking opioids, five taking benzodiazepines or zolpidem, and three taking duloxetine), and the low dosages used.

Finally, because blood draws were not conducted in this study, we did not obtain measures of peripheral inflammation such as C-reactive protein or erythrocyte sedimentation rate. It is possible that the correlations we observed between joint pain and brain metabolites were mediated by systemic inflammation. To fully understand the relationship of peripheral and central aspects of RA, future studies should collect measures of systemic inflammation.

In summary, while there are occasional reports of RA provoking severe central inflammation (such as rheumatoid meningitis), there is no direct evidence that brain inflammation is a typical feature of RA. The MRSI outcomes may be able to differentiate between patients with central versus peripheral causes of pain and fatigue, as we previously observed many MRSI abnormalities in chronic fatigue patients [14]. Much larger studies, including direct comparisons of medical conditions such as RA and fibromyalgia, would be required to develop a useful tool. We conclude that brain abnormalities of CHO, LAC, MI, and temperature are not characteristic of RA. Further research will be needed to determine if MRSI can help explain why some RA patients experience significant pain sensitivity and fatigue even when being medically managed.

## Electronic Supplementary Material

Online Resource 1 (PDF 40 kb)

Online Resource 2 (PDF 193 kb)

Online Resource 3 (PDF 63 kb)
