# Supplementary material for: No evidence of abnormal metabolic or inflammatory activity in the brains of patients with rheumatoid arthritis: results from a preliminary study using whole-brain magnetic resonance spectroscopic imaging (MRSI)
Source: Clin Rheumatol. 2020 Jan 30;39(6):1765–74. doi: 10.1007/s10067-019-04923-5 (PMC7237391; doi:10.1007/s10067-019-04923-5)
Supplement: Supplementary file 1 — (PDF 40 kb) [file 10067_2019_4923_MOESM1_ESM.pdf]

Clinical Rheumatology

No evidence of abnormal metabolic or inflammatory activity in the brains of patients with rheumatoid arthritis: examination via whole-brain magnetic resonance spectroscopic imaging (MRSI).

Christina Mueller, M.S., Joanne C. Lin, Ph.D., Halle H. Thannickal, Altamish Daredia, B.S., Thomas S. Denney, Ph.D., Ronald Beyers, Ph.D., Jarred W. Younger, Ph.D.

*Jarred W. Younger (corresponding author)
Department of Psychology, University of Alabama at Birmingham
Campbell Hall suite 233, 1300 University Blvd, Birmingham, AL 35233
e-mail: younger@uab.edu

Participant ID	Age	DMARDs	Other medications
RA003	30	hydroxychloroquine, etanercept	cymbalta, temazepam, diazepam, Mg, vitamin D, tramadol, cysta-Q, D-mannose,
RA004	39	infliximab	solumedrol, acetaminophen-oxycodone, alprazolam, duloxetine
RA006	24	adalimumab	Diclofenac
RA008	37	etanercept	nabumetone, acetaminophen-hydrocodone, zolpidem
RA009	35	methotrexate, adalimumab	meloxicam, docusate, iron supplement, folic acid
RA012	46	methotrexate, hydroxychloroquine, abatacept	prednisone, meloxicam, duloxetine, dexlansoprazole, alendronic acid, ranitidine, folic acid, vitamin B2, multivitamin
RA015	37	tocilizumab, hydroxychloroquine	acetaminophen-hydrocodone, insulin, levothyroxine, famotidine-ibuprofen, aspirin, vitamin E, calcium, alpha-lipoic acid, CoQ10
RA016	57	methotrexate	meloxicam, unspecified antihypertensive
RA017	61	abatacept, methotrexate	Pseudoephedrine, methocarbamol, montelukast, verapamil, tramadol, cyclosporine, omeprazole, hydrocodone-acetaminophen, zolpidem, progesterone, vitamin E, vitamin B12, folic acid, vitamin D, calcium, evening primrose
RA018	38	tocilizumab, methotrexate	atenolol, folic acid
RA019	39	methotrexate	meloxicam, folic acid
RA020	53		prednisone, flavocoxid, quinacrine, amitryptiline, propanolol, lorazepam, vitamin B complex, hydromorphone
RA022	35	Infliximab, sulfasalazine	none

Participants’ disease modifying anti-rheumatic drugs (DMARDs) as well as other medications taken regularly while participating in this study.
