# Supplementary material for: No evidence of abnormal metabolic or inflammatory activity in the brains of patients with rheumatoid arthritis: results from a preliminary study using whole-brain magnetic resonance spectroscopic imaging (MRSI)
Source: Clin Rheumatol. 2020 Jan 30;39(6):1765–74. doi: 10.1007/s10067-019-04923-5 (PMC7237391; doi:10.1007/s10067-019-04923-5)
Supplement: Supplementary file 2 — (PDF 193 kb) [file 10067_2019_4923_MOESM2_ESM.pdf]

Clinical Rheumatology

No evidence of abnormal metabolic or inflammatory activity in the brains of patients with rheumatoid arthritis: examination via whole-brain magnetic resonance spectroscopic imaging (MRSI).

Christina Mueller, M.S., Joanne C. Lin, Ph.D., Halle H. Thannickal, Altamish Daredia, B.S., Thomas S. Denney, Ph.D., Ronald Beyers, Ph.D., Jarred W. Younger, Ph.D.

*Jarred W. Younger (corresponding author)
Department of Psychology, University of Alabama at Birmingham
Campbell Hall suite 233, 1300 University Blvd, Birmingham, AL 35233
e-mail: younger@uab.edu

	RA			Healthy Controls					
**NAA/CR**	**N**	**Mean**	**SD**	**N**	**Mean**	**SD**	**t**	**df**	**p**
Precentral R	13	1.397	0.147	13	1.444	0.221	-0.637	24	0.530
Precentral L	13	1.401	0.137	13	1.477	0.265	-0.921	24	0.366
Frontal R	13	1.303	0.151	13	1.331	0.132	-0.498	24	0.623
Frontal L	13	1.370	0.216	13	1.347	0.151	0.309	24	0.760
Rolandic Oper R	13	1.272	0.135	13	1.230	0.100	0.914	24	0.370
Rolandic Oper L	13	1.267	0.144	13	1.260	0.112	0.148	24	0.884
Supp Motor Area R	12	1.219	0.155	13	1.406	0.436	-1.400	23	0.175
Supp Motor Area L	13	1.295	0.141	13	1.542	0.668	-1.303	24	0.205
Insula R	13	1.174	0.111	13	1.143	0.110	0.711	24	0.484
Insula L	13	1.177	0.157	13	1.114	0.094	1.253	24	0.222
Cingulum Ant R	12	1.167	0.150	13	1.135	0.194	0.458	23	0.651
Cingulum Ant L	13	1.200	0.185	13	1.105	0.120	1.555	24	0.133
Cingulum Mid R	13	1.227	0.148	13	1.295	0.267	-0.804	24	0.429
Cingulum Mid L	13	1.237	0.133	13	1.266	0.165	-0.482	24	0.634
Cingulum Post R	12	1.355	0.219	13	1.343	0.137	0.171	23	0.866
Cingulum Post L	13	1.507	0.241	13	1.484	0.121	0.305	17.651*	0.764
Hippocampus R	13	1.109	0.305	13	1.042	0.144	0.721	24	0.478
Hippocampus L	13	1.052	0.198	13	1.009	0.124	0.663	24	0.514
Calcarine R	13	1.323	0.144	13	1.357	0.170	-0.560	24	0.580
Calcarine L	13	1.373	0.155	13	1.449	0.216	-1.028	24	0.314
Cuneus R	13	1.308	0.137	13	1.397	0.159	-1.523	24	0.141
Cuneus L	13	1.382	0.165	13	1.489	0.280	-1.188	24	0.247
Lingual R	13	1.231	0.182	13	1.220	0.185	0.154	24	0.879
Lingual L	13	1.291	0.154	13	1.310	0.197	-0.274	24	0.786

Occipital R	13	1.290	0.135	13	1.365	0.185	-1.182	24	0.249
Occipital L	13	1.401	0.187	13	1.491	0.203	-1.169	24	0.254
Fusiform R	13	1.066	0.199	13	1.107	0.175	-0.558	24	0.582
Fusiform L	13	1.105	0.182	13	1.111	0.166	-0.086	24	0.932
Postcentral R	13	1.366	0.137	13	1.461	0.431	-0.760	24	0.455
Postcentral L	13	1.342	0.112	13	1.471	0.282	-1.532	24	0.139
Parietal R	13	1.289	0.158	13	1.390	0.309	-1.044	24	0.307
Parietal L	13	1.349	0.162	13	1.456	0.217	-1.415	24	0.170
Precuneus R	13	1.260	0.154	13	1.356	0.363	-0.876	24	0.390
Precuneus L	13	1.328	0.154	13	1.381	0.251	-0.649	24	0.522
Paracentral Lobule R	12	1.308	0.167	13	1.578	0.720	-1.269	23	0.217
Paracentral Lobule L	13	1.223	0.407	13	1.486	0.410	-1.638	24	0.114
Caudate R	13	1.224	0.154	13	1.190	0.122	0.625	24	0.538
Caudate L	13	1.252	0.281	13	1.170	0.150	0.926	24	0.363
Putamen R	13	1.171	0.159	13	1.133	0.105	0.700	24	0.491
Putamen L	13	1.138	0.137	13	1.070	0.087	1.521	24	0.141
Pallidum R	13	1.264	0.246	10	1.198	0.250	0.638	21	0.530
Pallidum L	13	1.215	0.323	12	0.996	0.154	2.129	23	0.044
Thalamus R	13	1.225	0.155	13	1.188	0.159	0.597	24	0.556
Thalamus L	13	1.222	0.178	13	1.173	0.100	0.867	24	0.395
Temporal R	13	1.191	0.121	13	1.197	0.094	-0.140	24	0.890
Temporal L	13	1.241	0.124	13	1.284	0.081	-1.064	24	0.298
Cerebellum	13	0.711	0.204	13	0.782	0.131	-1.050	24	0.304

	**RA**			**Healthy Controls**					
**CHO/CR**	**N**	**Mean**	**SD**	**N**	**Mean**	**SD**	**t**	**df**	**p**

Precentral R	13	0.184	0.025	13	0.183	0.022	0.080	24	0.937
Precentral L	13	0.176	0.024	13	0.173	0.019	0.263	24	0.795
Frontal R	13	0.193	0.024	13	0.191	0.021	0.273	24	0.787
Frontal L	13	0.188	0.022	13	0.188	0.019	0.033	24	0.974
Rolandic Oper R	13	0.195	0.027	13	0.191	0.019	0.487	24	0.631
Rolandic Oper L	13	0.184	0.027	13	0.192	0.021	-0.773	24	0.447
Supp Motor Area R	12	0.194	0.031	13	0.187	0.031	0.556	23	0.583
Supp Motor Area L	13	0.191	0.026	13	0.185	0.017	0.623	24	0.539
Insula R	13	0.219	0.028	13	0.219	0.019	0.032	24	0.975
Insula L	13	0.212	0.023	13	0.211	0.019	0.053	24	0.958
Cingulum Ant R	12	0.214	0.029	13	0.212	0.033	0.175	23	0.863
Cingulum Ant L	13	0.192	0.039	13	0.233	0.051	-2.305	24	0.030
Cingulum Mid R	13	0.201	0.025	13	0.195	0.031	0.567	24	0.576
Cingulum Mid L	13	0.204	0.029	13	0.205	0.026	-0.112	24	0.912
Cingulum Post R	12	0.199	0.033	13	0.186	0.024	1.140	23	0.266
Cingulum Post L	13	0.213	0.036	13	0.201	0.024	0.960	24	0.347
Hippocampus R	13	0.239	0.049	13	0.236	0.026	0.197	24	0.845
Hippocampus L	13	0.243	0.034	13	0.246	0.021	-0.302	20.178*	0.766
Calcarine R	13	0.156	0.027	13	0.147	0.022	0.952	24	0.350
Calcarine L	13	0.170	0.029	13	0.161	0.027	0.856	24	0.400
Cuneus R	13	0.143	0.022	13	0.144	0.023	-0.059	24	0.953
Cuneus L	13	0.147	0.024	13	0.151	0.029	-0.370	24	0.715
Lingual R	13	0.168	0.031	13	0.163	0.025	0.472	24	0.641
Lingual L	13	0.168	0.030	13	0.158	0.024	0.947	24	0.353
Occipital R	13	0.158	0.026	13	0.161	0.030	-0.215	24	0.831
Occipital L	13	0.151	0.036	13	0.156	0.037	-0.332	24	0.742

Fusiform R	13	0.183	0.040	13	0.183	0.029	0.026	24	0.979
Fusiform L	13	0.183	0.036	13	0.177	0.029	0.440	24	0.664
Postcentral R	13	0.172	0.025	13	0.167	0.025	0.526	24	0.604
Postcentral L	13	0.164	0.033	13	0.168	0.027	-0.323	24	0.749
Parietal R	13	0.168	0.026	13	0.165	0.024	0.341	24	0.736
Parietal L	13	0.157	0.034	13	0.164	0.029	-0.516	24	0.610
Precuneus R	13	0.164	0.029	13	0.164	0.021	0.037	24	0.971
Precuneus L	13	0.171	0.026	13	0.168	0.025	0.283	24	0.779
Paracentral Lobule R	12	0.180	0.034	13	0.172	0.015	0.734	14.696*	0.474
Paracentral Lobule L	13	0.177	0.034	13	0.169	0.022	0.70	24	0.491
Caudate R	13	0.233	0.044	13	0.230	0.040	0.185	24	0.854
Caudate L	13	0.241	0.046	13	0.212	0.023	1.977	17.645*	0.064
Putamen R	13	0.202	0.034	13	0.194	0.032	0.620	24	0.541
Putamen L	13	0.207	0.028	13	0.200	0.023	0.670	24	0.509
Pallidum R	13	0.183	0.045	10	0.186	0.040	-0.195	21	0.847
Pallidum L	13	0.179	0.064	12	0.161	0.055	0.781	23	0.443
Thalamus R	13	0.228	0.028	13	0.217	0.044	0.759	24	0.455
Thalamus L	13	0.224	0.029	13	0.215	0.025	0.831	24	0.414
Temporal R	13	0.183	0.028	13	0.181	0.022	0.173	24	0.864
Temporal L	13	0.175	0.031	13	0.179	0.022	-0.365	24	0.719
Cerebellum	13	0.197	0.034	13	0.194	0.030	0.231	24	0.819

	**RA**			**Healthy Controls**					
**MI/CR**	**N**	**Mean**	**SD**	**N**	**Mean**	**SD**	**t**	**df**	**p**
Precentral R	13	0.520	0.125	13	0.535	0.197	-0.244	24	0.810
Precentral L	13	0.543	0.085	13	0.506	0.090	1.067	24	0.297

Frontal R	13	0.560	0.097	13	0.513	0.110	1.168	24	0.254
Frontal L	13	0.554	0.082	13	0.562	0.116	-0.184	24	0.855
Rolandic Oper R	13	0.542	0.103	13	0.514	0.117	0.655	24	0.519
Rolandic Oper L	13	0.544	0.100	13	0.584	0.172	-0.736	24	0.469
Supp Motor Area R	12	0.460	0.115	13	0.496	0.111	-0.776	23	0.446
Supp Motor Area L	13	0.522	0.104	13	0.452	0.140	1.435	24	0.164
Insula R	13	0.550	0.144	13	0.595	0.189	-0.690	24	0.497
Insula L	13	0.570	0.151	13	0.593	0.105	-0.449	24	0.658
Cingulum Ant R	12	0.537	0.188	13	0.565	0.208	-0.360	23	0.722
Cingulum Ant L	13	0.722	0.329	13	0.556	0.236	1.481	24	0.152
Cingulum Mid R	13	0.560	0.084	13	0.570	0.100	-0.296	24	0.769
Cingulum Mid L	13	0.665	0.208	13	0.567	0.095	1.544	24	0.136
Cingulum Post R	12	0.581	0.114	13	0.569	0.107	0.266	23	0.792
Cingulum Post L	13	0.618	0.105	13	0.591	0.150	0.526	24	0.604
Hippocampus R	13	0.655	0.179	13	0.633	0.142	0.337	24	0.739
Hippocampus L	13	0.665	0.170	13	0.658	0.106	0.136	24	0.893
Calcarine R	13	0.538	0.092	13	0.520	0.090	0.502	24	0.620
Calcarine L	13	0.564	0.083	13	0.542	0.158	0.457	24	0.652
Cuneus R	13	0.516	0.159	13	0.513	0.101	0.063	24	0.950
Cuneus L	13	0.530	0.148	13	0.578	0.063	-1.088	16.176*	0.293
Lingual R	13	0.580	0.098	13	0.536	0.097	1.136	24	0.267
Lingual L	13	0.575	0.110	13	0.516	0.142	1.184	24	0.248
Occipital R	13	0.515	0.120	13	0.515	0.084	0.015	24	0.988
Occipital L	13	0.553	0.125	13	0.580	0.099	-0.615	24	0.544
Fusiform R	13	0.577	0.103	13	0.522	0.096	1.417	24	0.169
Fusiform L	13	0.549	0.136	13	0.527	0.135	0.404	24	0.690

Postcentral R	13	0.497	0.118	13	0.526	0.151	-0.549	24	0.588
Postcentral L	13	0.529	0.097	13	0.496	0.075	0.969	24	0.342
Parietal R	13	0.519	0.092	13	0.516	0.101	0.091	24	0.928
Parietal L	13	0.567	0.102	13	0.562	0.104	0.130	24	0.898
Precuneus R	13	0.521	0.089	13	0.504	0.135	0.389	24	0.701
Precuneus L	13	0.557	0.080	13	0.556	0.095	0.023	24	0.982
Paracentral Lobule R	12	0.465	0.145	13	0.464	0.140	0.014	23	0.989
Paracentral Lobule L	13	0.524	0.185	13	0.441	0.130	1.314	24	0.201
Caudate R	13	0.584	0.172	13	0.522	0.216	0.810	24	0.426
Caudate L	13	0.622	0.139	13	0.594	0.144	0.504	24	0.619
Putamen R	13	0.456	0.201	13	0.528	0.268	-0.783	24	0.441
Putamen L	13	0.492	0.172	13	0.479	0.111	0.228	24	0.822
Pallidum R	13	0.403	0.246	10	0.302	0.129	1.174	21	0.254
Pallidum L	13	0.413	0.474	12	0.396	0.228	0.114	23	0.910
Thalamus R	13	0.652	0.256	13	0.556	0.135	1.187	24	0.247
Thalamus L	13	0.583	0.164	13	0.591	0.175	-0.113	24	0.911
Temporal R	13	0.544	0.096	13	0.530	0.094	0.356	24	0.725
Temporal L	13	0.547	0.109	13	0.520	0.104	0.642	24	0.527
Cerebellum	13	0.529	0.103	13	0.475	0.090	1.423	24	0.168

	**RA**			**Healthy Controls**					
**LAC/CR**	**N**	**Mean**	**SD**	**N**	**Mean**	**SD**	**t**	**df**	**p**
Precentral R	13	0.313	0.299	13	0.181	0.181	1.358	24	0.187
Precentral L	13	0.247	0.258	13	0.193	0.184	0.619	24	0.542
Frontal R	13	0.349	0.171	13	0.403	0.375	-0.467	24	0.645
Frontal L	13	0.453	0.379	13	0.341	0.212	0.932	24	0.361

Rolandic Oper R	13	0.116	0.099	13	0.087	0.080	0.820	24	0.420
Rolandic Oper L	13	0.142	0.223	13	0.134	0.180	0.097	24	0.923
Supp Motor Area R	12	0.175	0.201	13	0.186	0.147	-0.155	23	0.879
Supp Motor Area L	13	0.235	0.238	13	0.183	0.183	0.628	24	0.536
Insula R	13	0.251	0.281	13	0.141	0.135	1.276	24	0.214
Insula L	13	0.232	0.270	13	0.166	0.153	0.768	24	0.450
Cingulum Ant R	12	0.228	0.167	13	0.287	0.340	-0.544	23	0.592
Cingulum Ant L	13	0.441	0.499	13	0.300	0.356	0.830	24	0.414
Cingulum Mid R	13	0.088	0.114	13	0.132	0.175	-0.748	24	0.462
Cingulum Mid L	13	0.202	0.317	13	0.163	0.225	0.365	24	0.718
Cingulum Post R	12	0.114	0.160	13	0.132	0.171	-0.271	23	0.789
Cingulum Post L	13	0.087	0.120	13	0.189	0.270	-1.245	24	0.225
Hippocampus R	13	0.199	0.178	13	0.114	0.061	1.632	14.774*	0.124
Hippocampus L	13	0.132	0.156	13	0.125	0.159	0.115	24	0.909
Calcarine R	13	0.174	0.098	13	0.236	0.233	-0.884	16.129*	0.390
Calcarine L	13	0.144	0.104	13	0.239	0.327	-1.000	24	0.327
Cuneus R	13	0.315	0.227	13	0.313	0.218	0.023	24	0.982
Cuneus L	13	0.254	0.203	13	0.276	0.248	-0.249	24	0.805
Lingual R	13	0.163	0.154	13	0.138	0.131	0.449	24	0.657
Lingual L	13	0.136	0.090	13	0.148	0.118	-0.284	24	0.779
Occipital R	13	0.386	0.215	13	0.364	0.188	0.288	24	0.776
Occipital L	13	0.321	0.178	13	0.317	0.164	0.050	24	0.960
Fusiform R	13	0.095	0.088	13	0.096	0.076	-0.032	24	0.975
Fusiform L	13	0.058	0.048	13	0.101	0.162	-0.909	24	0.372
Postcentral R	13	0.255	0.190	13	0.225	0.187	0.403	24	0.691
Postcentral L	13	0.241	0.281	13	0.229	0.276	0.111	24	0.912

Parietal R	13	0.278	0.182	13	0.251	0.194	0.368	24	0.716
Parietal L	13	0.188	0.172	13	0.228	0.181	-0.572	24	0.573
Precuneus R	13	0.170	0.122	13	0.225	0.205	-0.824	24	0.418
Precuneus L	13	0.111	0.083	13	0.213	0.223	-1.549	15.29*	0.142
Paracentral Lobule R	12	0.227	0.243	13	0.236	0.167	-0.111	23	0.913
Paracentral Lobule L	13	0.233	0.197	13	0.296	0.297	-0.633	24	0.532
Caudate R	13	0.345	0.281	13	0.401	0.514	-0.349	24	0.730
Caudate L	13	0.195	0.176	13	0.260	0.245	-0.777	24	0.445
Putamen R	13	0.247	0.212	13	0.126	0.118	1.793	18.778*	0.089
Putamen L	13	0.143	0.135	13	0.188	0.199	-0.668	24	0.510
Pallidum R	13	0.210	0.225	10	0.150	0.210	0.658	21	0.518
Pallidum L	13	0.056	0.077	12	0.100	0.140	-0.994	23	0.331
Thalamus R	13	0.165	0.170	13	0.133	0.118	0.558	24	0.582
Thalamus L	13	0.109	0.101	13	0.124	0.138	-0.318	24	0.754
Temporal R	13	0.207	0.124	13	0.195	0.120	0.238	24	0.814
Temporal L	13	0.154	0.119	13	0.208	0.152	-1.015	24	0.32
Cerebellum	13	0.126	0.103	13	0.112	0.081	0.373	24	0.712

	**RA**			**Healthy Controls**					
**Temperature**	**N**	**Mean**	**SD**	**N**	**Mean**	**SD**	**t**	**df**	**p**
Precentral R	13	5.094	0.372	13	5.023	0.429	0.453	24	0.654
Precentral L	13	4.963	0.386	13	5.019	0.413	-0.357	24	0.724
Frontal R	13	4.851	0.282	13	4.838	0.622	0.068	16.722*	0.947
Frontal L	13	4.970	0.227	13	4.868	0.577	0.594	15.625*	0.561
Rolandic Oper R	13	4.879	0.389	13	5.019	0.502	-0.796	24	0.434
Rolandic Oper L	13	4.834	0.383	13	4.930	0.456	-0.584	24	0.564

Supp Motor Area R	12	5.118	0.314	13	5.030	0.687	0.407	23	0.688
Supp Motor Area L	13	5.178	0.383	13	4.911	0.837	1.044	24	0.307
Insula R	13	4.602	0.320	13	4.647	0.650	-0.220	17.484*	0.828
Insula L	13	4.766	0.363	13	4.655	0.501	0.650	24	0.522
Cingulum Ant R	13	4.547	0.356	13	4.448	0.477	0.586	23	0.563
Cingulum Ant L	13	4.901	0.679	13	4.349	0.972	1.679	24	0.106
Cingulum Mid R	13	5.255	0.365	13	5.365	0.407	-0.722	24	0.477
Cingulum Mid L	13	5.266	0.340	13	5.328	0.449	-0.392	24	0.699
Cingulum Post R	12	5.101	0.487	13	5.221	0.390	-0.681	23	0.503
Cingulum Post L	13	5.037	0.382	13	5.134	0.459	-0.589	24	0.562
Hippocampus R	13	4.380	0.701	13	4.057	0.499	1.355	24	0.188
Hippocampus L	13	4.012	0.337	13	4.178	0.455	-1.060	24	0.300
Calcarine R	13	4.763	0.427	13	4.843	0.570	-0.406	24	0.689
Calcarine L	13	4.857	0.434	13	4.950	0.521	-0.497	24	0.624
Cuneus R	13	4.962	0.443	13	4.983	0.675	-0.094	24	0.926
Cuneus L	13	4.869	0.456	13	4.950	0.523	-0.423	24	0.676
Lingual R	13	4.874	0.340	13	4.825	0.446	0.320	24	0.752
Lingual L	13	4.732	0.345	13	4.809	0.394	-0.530	24	0.601
Occipital R	13	4.732	0.449	13	4.664	0.606	0.322	24	0.750
Occipital L	13	4.690	0.448	13	4.741	0.524	-0.265	24	0.793
Fusiform R	13	4.687	0.427	13	4.482	0.655	0.946	24	0.354
Fusiform L	13	4.524	0.541	13	4.660	0.641	-0.587	24	0.563
Postcentral R	13	4.967	0.384	13	5.023	0.466	-0.336	24	0.740
Postcentral L	13	4.960	0.278	13	5.080	0.391	-0.895	24	0.380
Parietal R	13	5.100	0.299	13	5.158	0.460	-0.386	24	0.703
Parietal L	13	5.029	0.281	13	5.275	0.428	-1.736	24	0.095

Precuneus R	13	5.268	0.346	13	5.316	0.490	-0.289	24	0.775
Precuneus L	13	5.232	0.323	13	5.314	0.375	-0.599	24	0.555
Paracentral Lobule R	12	4.937	0.526	13	5.110	0.646	-0.728	23	0.474
Paracentral Lobule L	13	4.713	1.278	13	5.028	0.645	-0.792	24	0.436
Caudate R	13	4.989	0.408	13	5.027	0.584	-0.190	24	0.851
Caudate L	13	4.864	0.461	13	4.858	0.501	0.036	24	0.972
Putamen R	13	4.214	0.529	13	4.281	0.576	-0.310	24	0.759
Putamen L	13	4.211	0.648	13	4.073	0.618	0.553	24	0.586
Pallidum R	13	3.937	0.899	10	3.495	0.535	1.375	21	0.184
Pallidum L	13	3.431	0.486	12	3.641	0.853	-0.766	23	0.451
Thalamus R	13	4.526	0.351	13	4.358	0.729	0.746	24	0.463
Thalamus L	13	4.533	0.445	13	4.551	0.794	-0.072	24	0.944
Temporal R	13	4.475	0.420	13	4.504	0.415	-0.178	24	0.860
Temporal L	13	4.586	0.319	13	4.546	0.543	0.226	24	0.823
Cerebellum	13	4.559	0.341	13	4.641	0.561	-0.454	19.805*	0.655

*degrees of freedom (df) have been adjusted for unequal variances between groups

Mean metabolite ratios and their standard deviations (SD) in the RA and healthy control groups. t- and p-values refer to independent-samples t-tests comparing metabolite ratios between the groups. None of the results survived corrections for multiple comparisons.
