# Supplementary material for: No evidence of abnormal metabolic or inflammatory activity in the brains of patients with rheumatoid arthritis: results from a preliminary study using whole-brain magnetic resonance spectroscopic imaging (MRSI)
Source: Clin Rheumatol. 2020 Jan 30;39(6):1765–74. doi: 10.1007/s10067-019-04923-5 (PMC7237391; doi:10.1007/s10067-019-04923-5)
Supplement: Supplementary file 3 — (PDF 63 kb) [file 10067_2019_4923_MOESM3_ESM.pdf]

Clinical Rheumatology

No evidence of abnormal metabolic or inflammatory activity in the brains of patients with rheumatoid arthritis: examination via whole-brain magnetic resonance spectroscopic imaging (MRSI).

Christina Mueller, M.S., Joanne C. Lin, Ph.D., Halle H. Thannickal, Altamish Daredia, B.S., Thomas S. Denney, Ph.D., Ronald Beyers, Ph.D., Jarred W. Younger, Ph.D.

*Jarred W. Younger (corresponding author)
Department of Psychology, University of Alabama at Birmingham
Campbell Hall suite 233, 1300 University Blvd, Birmingham, AL 35233
e-mail: younger@uab.edu

	LAC/CR			NAA/CR			Temperature (°C)		
**ROI**	**FSS VAS**	**DAS28 swollen**	**DAS28 tender**	**FSS VAS**	**DAS28 swollen**	**DAS28 tender**	**FSS VAS**	**DAS28 swollen**	**DAS28 tender**
Precentral R	0.008	0.023	-0.296	0.416	0.281	-0.033	0.323	-0.065	0.234
Precentral L	0.028	0.043	-0.195	0.098	0.385	0.081	-0.337	0.136	0.064
Frontal Lobe R	0.152	-0.113	-0.385	0.287	0.238	-0.139	-0.438	-0.156	-0.128
Frontal Lobe L	0.048	-0.094	-0.209	0.250	0.456	-0.020	-0.275	-0.054	-0.006
Rolandic Oper R	-0.208	0.238	-0.131	-0.093	0.130	-0.153	0.157	-0.371	0.064
Rolandic Oper L	-0.096	0.187	0.092	0.483	0.150	-0.028	0.166	0.283	0.287
Supp Motor Area R	0.128†	-0.018†	-0.363†	0.292†	0.174†	-0.113†	0.217†	0.054†	0.109†
Supp Motor Area L	0.065	0.051	-0.248	0.214	0.442	0.092	-0.128†	-0.109†	0.116†
Insula R	-0.118	-0.099	-0.195	0.219	0.329	0.000	-0.396	-0.326	-0.198
Insula L	-0.132	-0.196	-0.391	0.244	0.448	0.251	0.250	0.088	0.123
Anterior Cingulum R	0.142†	-0.454†	-0.589*†	0.292†	0.047†	0.201†	-0.089†	-0.011†	-0.254†
Anterior Cingulum L	-0.073	-0.425	-0.700**	0.306	-0.065	-0.114	-0.461	-0.207	-0.318
Mid Cingulum R	0.084	-0.235	-0.198	0.124	0.281	-0.039	0.050†	-0.229†	-0.014†
Mid Cingulum L	-0.239	-0.312	-0.494	0.230	0.142	-0.117	0.197	-0.128	0.061
Posterior Cingulum R	-0.101†	-0.118†	-0.203†	0.356†	0.050†	0.118†	0.029†	0.186†	0.285†
Posterior Cingulum L	0.135	0.020	-0.167	0.351	0.023	0.184	0.472	0.442	0.513

Hippocampus R	-0.126	-0.298	-0.449	0.346	0.247	0.262	-0.135†	-0.033†	0.233†
Hippocampus L	0.138	-0.045	-0.053	0.379	0.207	0.374	0.309	0.436	0.572*
Calcarine R	-0.135	-0.014	-0.192	0.340	-0.014	0.056	-0.073	-0.340	-0.014
Calcarine L	-0.348	-0.119	-0.117	-0.093	-0.003	-0.315	0.056	-0.391	0.056
Cuneus R	-0.402	-0.068	-0.304	0.396	0.266	0.086	0.129	-0.402	-0.028
Cuneus L	-0.129	-0.099	-0.050	-0.051	-0.048	-0.438	-0.031	-0.652*	-0.435
Lingual Gyrus R	-0.199	-0.303	-0.427	0.582*	0.218	0.416	0.045	-0.105	0.315
Lingual Gyrus L	-0.391	-0.383	-0.505	0.081	0.122	-0.095	0.037	-0.278	0.084
Occipital Lobe R	-0.126	-0.176	-0.145	0.028	0.320	0.067	0.253	0.048	0.008
Occipital Lobe L	-0.084	0.136	-0.268	-0.087	0.062	-0.352	-0.138	-0.612*	-0.379
Fusiform Gyrus R	0.081	-0.028	-0.134	0.205	0.170	0.259	0.219	-0.043	0.206
Fusiform Gyrus L	-0.051	-0.323	-0.170	0.385	0.317	0.232	0.334	-0.031	0.248
Postcentral Gyrus R	0.056	0.125	-0.075	0.104	0.241	-0.259	-0.233	-0.096	-0.128
Postcentral Gyrus L	0.160	-0.023	-0.279	-0.022	0.179	-0.089	-0.458	-0.224	-0.081
Parietal Lobe R	-0.171	0.227	0.047	0.157	0.266	-0.050	0.000	-0.162	-0.064
Parietal Lobe L	0.104	0.230	0.120	0.056	0.105	-0.273	-0.112	-0.470	-0.131
Precuneus R	-0.177	0.017	-0.310	0.169	0.374	0.081	0.112	-0.417	0.011
Precuneus L	-0.104	-0.130	-0.212	0.051	0.184	-0.114	-0.258	-0.536	-0.290
Paracentral Lobule R	0.064†	0.084†	-0.261†	0.470†	0.436†	0.113†	-0.037††	0.135††	0.221††

Paracentral Lobule L	0.214	0.043	-0.089	0.489	0.320	0.061	-0.353††	-0.308††	-0.133††
Caudate R	-0.281	-0.332	-0.335	0.208	0.130	0.117	-0.517	-0.570*	-0.469
Caudate L	-0.326	0.037	-0.237	-0.037	0.337	-0.109	-0.084	0.244	0.234
Putamen R	-0.140	-0.159	-0.312	0.332	-0.108	0.017	0.042	-0.315	-0.114
Putamen L	-0.149	-0.380	-0.402	0.079	0.249	0.059	0.199	0.136	0.120
Pallidum R	0.121	-0.051	-0.318	0.098	-0.326	-0.114	-0.278	-0.164	-0.293
Pallidum L	0.360	0.167	0.053	-0.275	-0.247	0.045	0.555†	0.055†	0.377†
Thalamus R	-0.205	-0.147	-0.159	0.469	0.349	0.379	-0.11	-0.128	0.006
Thalamus L	-0.020	0.150	0.151	0.129	0.30	0.170	0.107	0.153	0.234
Temporal Lobe R	-0.230	-0.298	-0.204	0.011	0.082	-0.075	0.242	-0.133	0.237
Temporal Lobe L	-0.107	-0.040	-0.117	0.132	-0.221	-0.262	0.017	-0.159	0.190
Cerebellum	-0.045	0.020	-0.229	-0.177	0.150	0.036	-0.112	-0.255	0.159

VAS: visual analog scale
*p<0.05 **p<0.01
n=13 (unless an exception is noted); †: n=12; ††: n=11

Spearman correlations between metabolite ratios and temperature in regions of interest and clinical outcome data in RA patients. None of the results survived corrections for multiple comparisons (equivalent to uncorrected p<0.0023).
